# Toxin genotypes and antimicrobial resistance profiles of *Clostridium perfringens* isolated from healthy and diseased goats in Jiangsu Province, China

**DOI:** 10.14202/vetworld.2026.409-421

**Published:** 2026-01-31

**Authors:** Zibei Huang, Siyuan Su, Haiyan Wang, Jinlin Huang, Wenbo Liu

**Affiliations:** 1 School of Veterinary Science, Yangzhou University, Yangzhou, China; 2 Jiangsu Vocational College of Agriculture and Animal Husbandry, Taizhou, China; 3 Key Laboratory of Prevention and Control of Biological Hazard Factors (Animal Origin) for Agrifood Safety and Quality, Ministry of Agriculture and Rural Affairs of China, Yangzhou, China

**Keywords:** antimicrobial resistance, *Clostridium perfringens*, goats, molecular epidemiology, One Health, PCR detection, toxin genotyping, veterinary microbiology

## Abstract

**Background and Aim:**

*Clostridium perfringens* is a major enteric pathogen of goats, capable of producing multiple toxins and harboring diverse antimicrobial resistance (AMR) determinants. The coexistence of toxin diversity and AMR complicates disease control and poses risks to animal health and antimicrobial stewardship. This study aimed to characterize toxin genotypes, phenotypic AMR patterns, and associated resistance genes in *C. perfringens* isolates obtained from healthy, diseased, and deceased goats in Jiangsu Province, China.

**Materials and Methods:**

A total of 404 samples were collected from goats between April 2021 and April 2022, including feces from healthy animals, rumen contents from slaughtered goats, and intestinal contents and visceral tissues from diseased or deceased goats. Isolation and identification of *C. perfringens* were performed using anaerobic culture and *16S rRNA* gene sequencing. Toxin genotyping targeting major toxin genes was conducted by polymerase chain reaction (PCR). Antimicrobial susceptibility was assessed using the Kirby–Bauer disk diffusion method, and resistance genes were detected by PCR. Hierarchical clustering was used to explore relationships between toxinotypes and AMR gene profiles.

**Results:**

Ninety-four *C. perfringens* isolates were recovered (23.3%). Toxinotype A predominated overall (61.7%) and was dominant among healthy goats, whereas toxinotypes D (52.9%), C (14.7%), and F (14.7%) were more frequently detected in diseased or deceased animals. High phenotypic resistance was observed to aminoglycosides, including kanamycin (72.3%), neomycin (66.0%), and gentamicin (58.5%), as well as trimethoprim–sulfamethoxazole (61.7%). All isolates remained susceptible to vancomycin, with low resistance to β-lactams. Resistance genes associated with aminoglycosides, sulfonamides, tetracyclines, quinolones, and lincosamides were widely distributed. Notably, the quinolone resistance gene *qnrS* and tetracycline resistance genes *tetA(P)* and *tetB(P)* were significantly more prevalent in isolates from diseased goats, particularly toxinotype D.

**Conclusion:**

Goat-associated *C. perfringens* in Jiangsu Province exhibits substantial toxin diversity and a high burden of AMR, with distinct differences between healthy and diseased animals. These findings underscore the need for continuous molecular surveillance, rational antimicrobial use, and integrated control strategies to mitigate risks to goat health and productivity within a One Health framework.

## INTRODUCTION

*Clostridium perfringens* is an anaerobic, Gram-positive, spore-forming bacterium and one of the most frequently encountered pathogens affecting goats and other livestock. It commonly inhabits intestinal contents, feces, and ruminal fluid [1–5] and is a major etiological agent of necrotic enteritis in goats, with type D enterotoxemia being particularly important due to its strong association with sudden death [6–8]. *C. perfringens* is capable of producing up to 23 toxins [9, 10] and, based on six major toxins, CPA (α), CPB (β), ETX (ε), Iota (ι), CPE, and NetB, strains are classified into seven toxinotypes (A–G) [11–14]. In addition, several non-typing toxins, including CPB2, PFO, and NetF [15], as well as BEC [16, 17], further contribute to its pathogenic potential [18]. The extensive use of antibiotics for growth promotion and disease prevention in livestock production has accelerated the global emergence of antimicrobial resistance (AMR) in *C. perfringens* [19, 20]. Prolonged and indiscriminate antimicrobial use facilitates the dissemination of resistant bacteria and resistance genes, posing a growing threat to both animal and public health [21].

Despite its clinical and economic importance, information on the molecular epidemiology of *C. perfringens* in goats remains limited [22, 23], particularly with respect to large-scale surveillance in Jiangsu Province, China, one of the country’s major goat-producing regions. Although studies from other Chinese provinces, such as Sichuan and Shandong, as well as from neighboring countries including India and Pakistan, have reported variable prevalence rates and toxinotype distributions in goats and sheep, comprehensive and systematic data focusing specifically on goats in China are still scarce. Moreover, knowledge regarding the distribution of AMR genes and their potential associations with toxinotypes in both healthy and diseased goats remains inadequate [24, 25].

Although *C. perfringens* is recognized as a major enteric pathogen of goats, comprehensive data on its molecular epidemiology in caprine populations remain limited, particularly in China. Most available studies have focused on sheep or poultry, involved small sample sizes, or were restricted to clinical cases, thereby overlooking the role of healthy goats as potential reservoirs of toxigenic and antimicrobial-resistant strains. In Jiangsu Province, one of the major goat-producing regions in China, systematic, large-scale investigations addressing toxinotype distribution, antimicrobial susceptibility patterns, and the underlying resistance genes of *C. perfringens* are scarce. Moreover, the relationship between toxin genotypes and AMR profiles in isolates derived from healthy versus diseased or deceased goats has not been sufficiently explored. This lack of integrated phenotypic and genotypic data hampers risk assessment, evidence-based antimicrobial stewardship, and the development of targeted prevention strategies within a One Health framework.

Therefore, the present study was designed to investigate the molecular epidemiology of *C. perfringens* isolated from healthy, diseased, and deceased goats in Jiangsu Province, China. Specifically, the study aimed to (i) determine the prevalence and toxinotype distribution of *C. perfringens* among different goat populations, (ii) characterize the phenotypic AMR profiles of the isolates, (iii) identify key AMR genes using polymerase chain reaction–based assays, and (iv) assess the association between toxin genotypes, resistance phenotypes, and resistance gene carriage. By integrating microbiological, molecular, and epidemiological approaches, this study provides baseline data to support improved disease control, rational antimicrobial use, and surveillance of toxigenic and antimicrobial-resistant *C. perfringens* in goat production systems.

## MATERIALS AND METHODS

### Ethical approval

The study protocol was reviewed and approved by the Laboratory Animal Ethics Committee of Yangzhou University (approval code: 202104051, dated April 15, 2021).

Fecal samples were collected noninvasively from clinically healthy goats, and clinical and postmortem specimens (intestinal contents and scrapings, rumen contents, and visceral tissues) were obtained from animals presented to the Veterinary Teaching Hospital of Yangzhou University or from routine slaughter at an abattoir. Samples were collected by trained personnel using aseptic techniques, with efforts to minimize animal stress and avoid unnecessary handling. Goats that had received antimicrobial treatment within 2 weeks before sampling were excluded from the “healthy” group to avoid confounding.

All sampling and laboratory procedures were conducted in accordance with biosafety practices appropriate for anaerobic enteric pathogens (BSL-2 conditions). Samples were transported on ice and processed within 12 h to maintain specimen integrity. Data were handled confidentially, and farm/owner identities were not disclosed in reporting.

### Study period and location

Sample collection was conducted between April 2021 and April 2022 at goat farms, slaughterhouses, and the Veterinary Teaching Hospital of Yangzhou University. This study was conducted in Jiangsu Province, China, a major goat-producing region characterized by a humid subtropical climate. Goat production systems in this region are predominantly small- to medium-scale, with animals raised under semi-intensive or free-range management conditions.

### Sample collection and classification

A total of 404 samples were collected from goats with different health statuses. Fresh fecal samples (n = 212) were obtained from clinically healthy goats reared on farms located in Taizhou (n = 40), Nantong (n = 45), Lianyungang (n = 45), Yancheng (n = 42), and Suzhou (n = 40). Goats from different age groups, kids (<6 months), yearlings (6–12 months), and adults (>12 months), were included to ensure age diversity, although detailed age information was not recorded for each individual animal. Healthy goats were defined as animals showing no clinical signs of disease, and goats that had received antibiotic treatment within two weeks prior to sampling were excluded.

Rumen content samples (n = 30) were collected from freshly slaughtered goats at a local abattoir in Lianyungang. Samples obtained from clinically healthy live goats and slaughtered goats were grouped together and categorized as “healthy” for subsequent analyses.

Clinical samples (n = 162), including intestinal contents, intestinal scrapings, rumen contents, and visceral tissues (liver, spleen, lungs, and kidneys), were collected from diseased or deceased goats submitted to the Veterinary Teaching Hospital of Yangzhou University. These animals originated from goat farms across multiple regions of Jiangsu Province, including Yangzhou, Taizhou, Yancheng, Nantong, Suzhou, Haian, and Yangzhong. Diseased or deceased goats were identified based on clinical signs such as diarrhea, enterotoxemia, sudden death, or other gastrointestinal manifestations, with *C. perfringens* infection suspected by field veterinarians based on clinical examination and postmortem findings.

### Sample handling and transport

**Figure 1:**
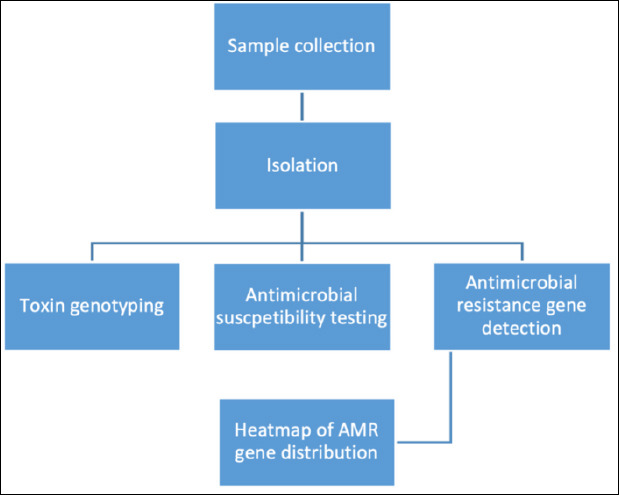
Flow diagram illustrating the study design, sample collection, classification of goats based on health status, and laboratory workflow for the isolation, identification, toxinotyping, and antimicrobial resistance analysis of *Clostridium perfringens* isolates.

Feces, rumen contents, and tissue samples were collected because *C. perfringens* colonizes the gastrointestinal tract and can disseminate to visceral organs, making these sites suitable for detecting both asymptomatic carriage and clinical infection. All samples were aseptically collected by trained personnel using sterile cotton swabs or disposable instruments, placed immediately into leak-proof containers, and stored on ice at 4°C during transport. Sample handling and laboratory processing were performed under Biosafety Level 2 (BSL-2) conditions. The maximum interval between sample collection and laboratory analysis did not exceed 12 h. The overall workflow of sample collection, transport, and processing is illustrated in Figure 1.

### Clinical observations of diseased or deceased goats

Clinical observations were recorded during field investigations and hospital admissions. Diseased or deceased goats commonly exhibited signs including anorexia, diarrhea, abdominal pain, respiratory distress, or sudden death prior to sampling. These observations supported the clinical suspicion of *C. perfringens* infection.

### Isolation and Identification of *C. perfringens*

Samples were initially inoculated into fluid thioglycollate (FTG) medium (Qingdao Hope Bio-Technology Co., Ltd., Qingdao, China) for anaerobic enrichment and heat-treated at 80 °C for 30 min to eliminate non-spore-forming bacteria. Cultures were then incubated anaerobically at 37°C for 24 h. Enriched samples were subcultured onto tryptose sulfite cycloserine agar (Qingdao Hope Bio-Technology Co., Ltd., Qingdao, China) and incubated anaerobically at 37°C for 24–48 h using a GasPak™ system (BD Diagnostics, Sparks, MD, USA), with anaerobic conditions verified by indicator strips.

Colonies exhibiting typical black pigmentation were presumptively identified as *C. perfringens* and subcultured at least three times to obtain pure isolates. Genomic DNA was extracted by boiling bacterial suspensions at 100°C for 10 min. Species confirmation was performed using polymerase chain reaction (PCR) amplification and 16S rRNA gene sequencing. *C. perfringens* ATCC 13124 served as the positive control.

### DNA extraction and PCR-based toxin genotyping

Genomic DNA was extracted from purified isolates using the boiling method. Briefly, bacterial colonies were suspended in sterile distilled water, boiled at 100°C for 10 min, and centrifuged at 12,000 × *g* for 5 min. The supernatant containing genomic DNA was collected and stored at −20°C until use.

PCR assays targeting the *cpa*, *cpb*, *etx*, *iap*, *cpe*, and *netB* toxin genes were performed using primers and protocols described in previous studies (Supplementary data). Toxinotypes were determined based on specific gene combinations: type A (*cpa*), type B (*cpa*, *cpb*, *etx*), type C (*cpa*, *cpb*, *cpe* positive or negative), type D (*cpa*, *etx*, *cpe* positive or negative), type E (*cpa*, *iap*, *cpe* positive or negative), type F (*cpa*, *cpe*), and type G (*cpa*, *netB*).

Each PCR reaction (25 μL) contained template DNA, gene-specific primers, deoxynucleotide triphosphates, Taq DNA polymerase, and reaction buffer. Amplified products were separated on 1.0% agarose gels stained with ethidium bromide, visualized under UV illumination, and documented using a gel imaging system. Previously confirmed *C. perfringens* isolates representing toxinotypes D, F, and G were used as positive controls, while nuclease-free water served as the negative control.

### Antimicrobial susceptibility testing

Antimicrobial susceptibility testing was performed using 30 antibiotic disks (Hangzhou Microbial Reagent Co., Ltd., Hangzhou, China). A complete list of antibiotics and disk concentrations is provided in the supplementary data. Susceptibility testing was conducted using the disk diffusion method in accordance with the Clinical and Laboratory Standards Institute guidelines (CLSI, M100-S24). As CLSI breakpoints specific for *C. perfringens* are not available, interpretative criteria for *Staphylococcus aureus* were applied as surrogate standards. The limitations of this approach were acknowledged, and results were interpreted cautiously.

*C. perfringens* ATCC 13124 was used as the reference strain for quality control. All antimicrobial susceptibility tests were performed in triplicate to ensure accuracy and reproducibility.

### Detection of AMR genes

DNA templates were prepared using the boiling method as described above. PCR assays targeting AMR genes were conducted using primers selected from previously published studies. Each 25 μL reaction mixture contained template DNA, specific primers, deoxynucleotide triphosphates, Taq DNA polymerase, PCR buffer, and nuclease-free water. Positive and negative controls were included in every PCR run.

Thermal cycling conditions consisted of an initial denaturation at 94°C for 5 min, followed by 30 cycles of denaturation at 94°C for 30 s, annealing at 52°C–60°C (depending on the primer set) for 30 s, and extension at 72°C for 1 min, with a final extension at 72°C for 7 min. Primer sequences and annealing temperatures are provided in the Additional Files.

PCR products were resolved on 1.0% agarose gels containing ethidium bromide and electrophoresed at 100 V for 30–40 min. Bands were visualized under UV light and photographed using a gel documentation system. DNA from previously confirmed resistant isolates served as positive controls, and nuclease-free water was used as the negative control.

### Statistical analysis

*16S rRNA* gene sequences were compared with reference sequences in the GenBank database using the Basic Local Alignment Search Tool. The presence or absence of toxin genes and AMR genes was summarized in tabular form. Prevalence and resistance rates were calculated as percentages with 95% confidence intervals.

Data visualization, including hierarchical clustering heat maps, was performed using OriginPro 2021 (OriginLab, Northampton, MA, USA). Hierarchical clustering employed Euclidean distance metrics with average linkage. Where applicable, statistical comparisons between isolates from healthy and diseased goats were conducted, with statistical significance defined as p < 0.05. Chi-square and Fisher’s exact tests were not applied to heatmap-based clustering analyses.

## RESULTS

### Prevalence and toxinotype distribution of *C. perfringens*

A total of 94 *C. perfringens* isolates (23.27%) were confirmed by *16S rRNA* gene sequencing. Overall, toxinotype A predominated, accounting for 61.70% (58/94) of isolates, followed by toxinotype D (20.21%, 19/94), toxinotype F (9.57%, 9/94), and toxinotype C (5.32%, 5/94).

Among isolates recovered from diseased or deceased goats, toxinotype D was the most prevalent (52.94%, 18/34), followed by toxinotype A (17.65%, 6/34), while toxinotypes C and F were each detected in 14.71% (5/34) of isolates. In contrast, fecal samples from healthy goats were dominated by toxinotype A (87.93%, 51/58), with lower frequencies of toxinotype F (6.90%, 4/58), toxinotype G (3.45%, 2/58), and toxinotype D (1.72%, 1/58); toxinotype C was not detected in this group. From rumen contents of slaughtered goats, only one isolate each of toxinotypes A and G was identified. Detailed toxinotype distributions according to sample source are summarized in Table 1.

**Table 1:** Prevalence and toxinotype distribution of *Clostridium perfringens* isolated from different sample types obtained from healthy and diseased/deceased goats in Jiangsu Province, China.

Group	Sample	Positive/Total	A	B	C	D	E	F	G
**Diseased**	Liver	4/27	1	0	0	2	0	1	0
	Lung	4/27	0	0	1	2	0	1	0
	Kidney	5/27	0	0	0	4	0	1	0
	Spleen	1/27	0	0	0	1	0	0	0
	Small intestine	9/27	2	0	1	5	0	1	0
	Appendix	11/27	3	0	3	4	0	1	0
	**Total**	**34/162**	**6 (17.65)**	**0**	**5 (14.71)**	**18 (52.94)**	**0**	**5 (14.71)**	**0**
**Healthy**	Stool	58/212	51	0	0	1	0	4	2
**Slaughtered**	Rumen contents	2/30	1	0	0	0	0	0	1
	**Total**	**60/242**	**52 (86.67)**	**0**	**0**	**1 (1.67)**	**0**	**4 (6.67)**	**3 (5.00)**
**Overall total**	—	**94/404**	**58 (61.70)**	**0**	**5 (5.32)**	**19 (20.21)**	**0**	**9 (9.57)**	**3 (3.19)**

Values under toxinotypes (A–G) are number of isolates; percentages in parentheses represent the proportion within each subtotal. Toxinotypes were determined based on the presence of major toxin genes detected by polymerase chain reaction. Percentages may not total 100 due to rounding.

### Phenotypic antimicrobial susceptibility profiles

Antimicrobial susceptibility testing was performed on 34 isolates from diseased or deceased goats and 20 isolates from healthy goats (Table 2). High levels of resistance were observed against aminoglycosides, including amikacin (90.74%), gentamicin (94.44%), kanamycin (98.15%), and neomycin (98.15%), as well as against the sulfonamide combination sulfamethoxazole–trimethoprim (98.15%).

**Table 2:** Phenotypic antimicrobial resistance patterns of *Clostridium perfringens* isolates recovered from healthy and diseased/deceased goats.

Antibiotic class	Antibiotic	Diseased (n = 34) R/n (%)	95% CI	Healthy (n = 20) R/n (%)	95% CI	p-value	Total (n = 54) R/n (%)	95% CI
Sulfanilamide	Trimethoprim–sulfamethoxazole	33/34 (97.1)	84.7–99.9	20/20 (100)	83.2–100	1.000	53/54 (98.1)	90.1–99.9
Lincosamide	Clindamycin	16/34 (47.1)	30.9–63.5	4/20 (20.0)	6.8–40.7	0.0789	20/54 (37.0)	25.0–50.9
Cephalosporins	Cephalexin	5/34 (14.7)	6.4–30.1	0/20 (0.0)	0.0–16.1	0.145	5/54 (9.3)	4.0–19.9
	Cefazolin	0/34 (0.0)	0.0–10.2	0/20 (0.0)	0.0–16.1	1.000	0/54 (0.0)	0.0–6.6
	Cephradine	5/34 (14.7)	6.4–30.1	0/20 (0.0)	0.0–16.1	0.145	5/54 (9.3)	4.0–19.9
	Cefuroxime	3/34 (8.8)	3.0–23.0	0/20 (0.0)	0.0–16.1	0.287	3/54 (5.6)	1.9–15.1
	Ceftazidime	6/34 (17.7)	8.3–33.5	0/20 (0.0)	0.0–16.1	0.0739	6/54 (11.1)	5.2–22.2
	Ceftriaxone	5/34 (14.7)	6.4–30.1	0/20 (0.0)	0.0–16.1	0.145	5/54 (9.3)	4.0–19.9
	Cefoperazone	0/34 (0.0)	0.0–10.2	1/20 (5.0)	0.9–23.6	0.370	1/54 (1.9)	0.3–9.8
Nitrofuran	Furazolidone	2/34 (5.9)	1.6–19.1	4/20 (20.0)	6.8–40.7	0.179	6/54 (11.1)	5.2–22.2
Amphenicol	Chloramphenicol	8/34 (23.5)	12.4–40.0	0/20 (0.0)	0.0–16.1	0.0201	8/54 (14.8)	7.7–26.6
Polypeptide	Vancomycin	0/34 (0.0)	0.0–10.2	0/20 (0.0)	0.0–16.1	1.000	0/54 (0.0)	0.0–6.6
	Polymyxin B	31/34 (91.2)	76.3–97.7	17/20 (85.0)	62.1–95.3	0.658	48/54 (88.9)	77.4–95.1
Macrolide	Erythromycin	18/34 (52.9)	35.1–70.1	3/20 (15.0)	5.2–36.0	0.00871	21/54 (38.9)	26.3–53.0
	Midecamycin	18/34 (52.9)	35.1–70.1	2/20 (10.0)	2.8–30.1	0.00290	20/54 (37.0)	25.0–50.9
Quinolones	Norfloxacin	16/34 (47.1)	30.9–63.5	4/20 (20.0)	6.8–40.7	0.0789	20/54 (37.0)	25.0–50.9
	Ofloxacin	12/34 (35.3)	21.5–52.4	2/20 (10.0)	2.8–30.1	0.0556	14/54 (25.9)	16.1–38.9
	Ciprofloxacin	15/34 (44.1)	28.1–61.4	2/20 (10.0)	2.8–30.1	0.0141	17/54 (31.5)	20.9–44.7
Aminoglycoside	Amikacin	34/34 (100)	89.9–100	1/20 (5.0)	0.9–23.6	1.09e-13	35/54 (64.8)	51.0–76.6
	Gentamicin	34/34 (100)	89.9–100	1/20 (5.0)	0.9–23.6	1.09e		

Results are expressed as number of resistant isolates/number tested, with percentages and 95% confidence intervals (CI) in brackets. Antimicrobial susceptibility was determined using the disk diffusion method. Interpretive criteria were applied according to Clinical and Laboratory Standards Institute guidelines using surrogate breakpoints due to the absence of species-specific criteria for Clostridium perfringens. Comparisons between healthy and diseased groups were performed where applicable. p < 0.05 = Statistically significant, 0.05 ≤ p < 0.1 = Marginally significant.

Isolates from healthy goats were fully susceptible to all tested penicillins and cephalosporins (0% resistance). In contrast, isolates from diseased or deceased goats exhibited resistance to penicillins (up to 23.53%) and cephalosporins (up to 17.65%). Partial susceptibility was noted for tetracyclines (57.41%), macrolides (37.97%), quinolones (31.48%), and polymyxin B (88.89%). Notably, all isolates remained susceptible to vancomycin, with no resistance detected.

### Distribution of AMR genes

PCR analysis revealed the presence of AMR genes associated with β-lactams, sulfonamides, aminoglycosides, macrolides, tetracyclines, quinolones, amphenicols, and lincosamides among the 94 *C. perfringens* isolates, with detection frequencies ranging from 3.19% to 11.17%. The most frequently detected genes included *bla*_TEM_ (14.89%), *aph(3’)-III-F* (10.64%), *tetA(P)* (23.40%), *tetB(P)* (19.15%), *qnrS* (15.96%), and *lnu(B)* (21.28%).

Sulfonamide resistance genes *sul1* and *sul3*, as well as the phenicol resistance gene *floR*, were not detected in isolates from healthy goats but were present at relatively high frequencies in isolates from diseased or deceased animals. The quinolone resistance gene *qnrS* was particularly prevalent among clinical isolates, detected in 44.12% of this group.

Comparative analysis demonstrated significantly higher detection rates of *aph(3’)-III-F* and *lnu(B)* in isolates from diseased or deceased goats than in those from healthy animals. Specifically, *aph(3’)-III-F* was detected in only one of 60 healthy-goat isolates, compared with nine of 34 isolates from diseased or deceased goats. Similarly, *lnu(B)* was identified in 35.29% of clinical isolates, approximately threefold higher than the 13.33% observed among isolates from healthy goats. Detailed distributions of resistance genes are presented in Table 3.

**Table 3:** Distribution of antimicrobial resistance genes detected by polymerase chain reaction in *Clostridium perfringens* isolates from healthy and diseased/deceased goats.

Antibiotic class	Gene	Clinical R/n (%)	95% CI	Healthy R/n (%)	95% CI	p-value	Total R/n (%)	95% CI
**β-Lactam**	*bla*_CTX-M_	0/34 (0.00)	0.00–10.0	0/60 (0.00)	0.00–6.02	–	0/94 (0.00)	0.00–3.95
	*bla*_SHV_	1/34 (2.94)	0.52–14.92	0/60 (0.00)	0.00–6.02	0.38	1/94 (1.06)	0.19–5.77
	*bla*_TEM_	4/34 (11.76)	4.67–26.62	10/60 (16.67)	9.27–28.01	0.77	14/94 (14.89)	9.02–23.64
**Sulfonamide**	*sul1*	4/34 (11.76)	4.67–26.62	0/60 (0.00)	0.00–6.02	0.02	4/94 (4.26)	1.67–10.49
	*sul2*	0/34 (0.00)	0.00–10.0	0/60 (0.00)	0.00–6.02	–	0/94 (0.00)	0.00–3.95
	*sul3*	3/34 (8.82)	3.05–23.04	0/60 (0.00)	0.00–6.02	0.06	3/94 (3.19)	1.09–8.95
**Aminoglycoside**	*aac(6′)/aph(2″)*	0/34 (0.00)	0.00–10.0	1/60 (1.67)	0.29–8.86	1.00	1/94 (1.06)	0.19–5.77
	*aph(3′)-III-F*	9/34 (26.47)	14.60–43.12	1/60 (1.67)	0.29–8.86	0.001	10/94 (10.64)	5.87–18.44
	*ant(4′,4″)*	1/34 (2.94)	0.52–14.92	0/60 (0.00)	0.00–6.02	0.38	1/94 (1.06)	0.19–5.77
**Tetracyclines**	*tetM*	1/34 (2.94)	0.52–14.92	4/60 (6.67)	2.62–15.93	0.67	5/94 (5.32)	2.31–11.79
	*tetA(P)*	8/34 (23.53)	12.44–40.00	14/60 (23.33)	14.44–35.44	1.00	22/94 (23.40)	15.86–33.16
	*tetB(P)*	9/34 (26.47)	14.60–43.12	9/60 (15.00)	8.10–26.11	0.18	18/94 (19.15)	12.40–28.36
	*tet(L)*	0/34 (0.00)	0.00–10.0	1/60 (1.67)	0.29–8.86	1.00	1/94 (1.06)	0.19–5.77
	*tet(K)*	1/34 (2.94)	0.52–14.92	1/60 (1.67)	0.29–8.86	1.00	2/94 (2.13)	0.59–7.39
	*tet(O)*	0/34 (0.00)	0.00–10.0	1/60 (1.67)	0.29–8.86	1.00	1/94 (1.06)	0.19–5.77
	*tetP(B)*	3/34 (8.82)	3.05–23.04	1/60 (1.67)	0.29–8.86	0.29	4/94 (4.26)	1.67–10.49
	*tet(W)*	0/34 (0.00)	0.00–10.0	0/60 (0.00)	0.00–6.02	–	0/94 (0.00)	0.00–3.95
**Macrolides**	*erm(A)*	2/34 (5.88)	1.63–19.09	4/60 (6.67)	2.62–15.93	1.00	6/94 (6.38)	2.97–13.23
	*erm(B)*	4/34 (11.76)	4.67–26.62	4/60 (6.67)	2.62–15.93	0.46	8/94 (8.51)	4.39–15.92
	*erm(C)*	0/34 (0.00)	0.00–10.0	0/60 (0.00)	0.00–6.02	–	0/94 (0.00)	0.00–3.95
**Quinolones**	*qnrS*	15/34 (44.12)	28.88–60.55	0/60 (0.00)	0.00–6.02	<0.001	15/94 (15.96)	9.85–24.99
	*aac(6′)-Ib*	1/34 (2.94)	0.52–14.92	0/60 (0.00)	0.00–6.02	0.38	1/94 (1.06)	0.19–5.77
**Amphenicol**	*floR*	3/34 (8.82)	3.05–23.04	0/60 (0.00)	0.00–6.02	0.06	3/94 (3.19)	1.09–8.95
**Lincosamide**	*lnu(A)*	1/34 (2.94)	0.52–14.92	0/60 (0.00)	0.00–6.02	0.38	1/94 (1.06)	0.19–5.77
	*lnu(B)*	12/34 (35.29)	21.49–52.09	8/60 (13.33)	6.91–24.16	0.03	20/94 (21.28)	14.14–30.70

Values represent the number of gene-positive isolates/total isolates examined, with percentages and 95% confidence intervals (CI) in brackets. Resistance genes were detected using polymerase chain reaction assays targeting β-lactam, aminoglycoside, tetracycline, macrolide, quinolone, sulfonamide, phenicol, and lincosamide resistance determinants. Statistical significance between groups was assessed where applicable. p < 0.05 = Statistically significant, 0.05 ≤ p < 0.1 = Marginally significant.

### Association between toxinotypes and AMR determinants

**Figure 2:**
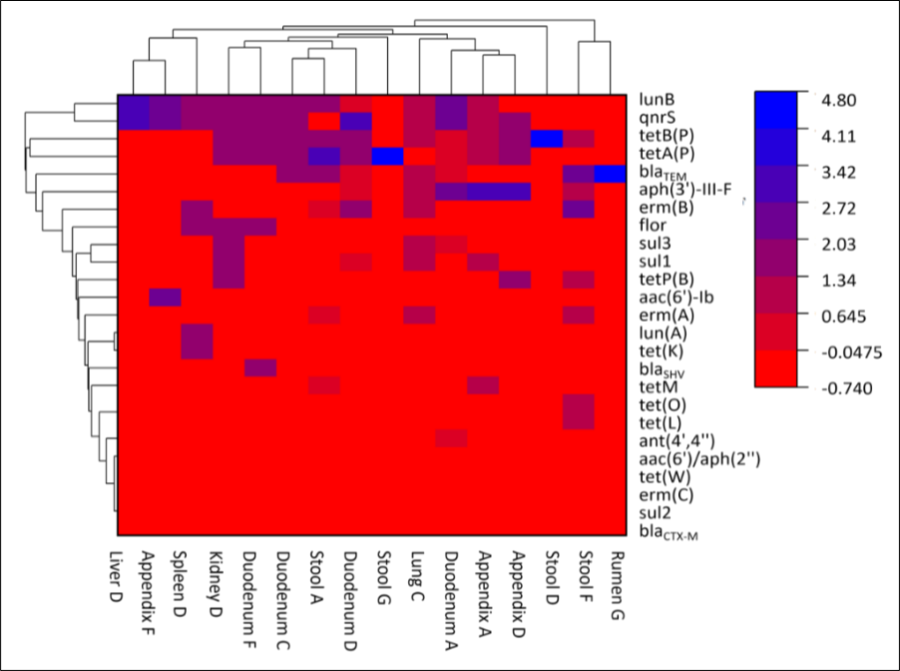
Heatmap showing the distribution of antimicrobial resistance genes in relation to toxinotypes of *Clostridium perfringens* isolates recovered from healthy and diseased/deceased goats. Rows represent individual isolates grouped by health status and toxinotype, while columns represent detected antimicrobial resistance genes. Color intensity indicates the presence of resistance determinants, with darker shading reflecting a higher number of detected genes. The heatmap is descriptive and does not imply causal relationships between toxinotypes and resistance profiles.

Hierarchical cluster analysis based on heatmap visualization was used to explore the relationship between toxinotypes and AMR gene profiles (Figure 2). Isolates derived from diseased or deceased goats generally exhibited higher frequencies of resistance to aminoglycosides, quinolones, and lincosamides compared with isolates from healthy goats.

Toxinotypes A and F from healthy goats displayed a broader diversity of resistance genes, whereas toxinotype D isolates from diseased or deceased animals harbored a higher number of resistance determinants, particularly the quinolone resistance gene *qnrS* and the tetracycline resistance genes *tetA(P)* and *tetB(P)*. These findings suggest that certain toxinotypes may have an enhanced capacity to acquire or retain multiple resistance genes, potentially reflecting differences in pathogenicity or prior antimicrobial exposure.

The diversity of resistance genes detected among isolates from healthy goats underscores the role of commensal *C. perfringens* populations as reservoirs of AMR, facilitating dissemination within herds. Overall, the clustering analysis reinforces the association between toxinotype and resistance profile and highlights the importance of integrated surveillance of both clinical and healthy goat populations to support effective antimicrobial stewardship strategies.

## DISCUSSION

### Distribution of *C. perfringens* toxinotypes in goats

Studies characterizing circulating *C. perfringens* toxinotypes in goats remain limited [26]. As a major foodborne pathogen and an important microorganism in medicine, veterinary science, and food safety, *C. perfringens* is a leading cause of enteric diseases, collectively referred to as enterotoxemia, in goats, sheep, and other livestock, resulting in substantial economic losses [27]. In the present study, we conducted a comprehensive investigation of 404 samples and identified 94 *C. perfringens* isolates from goats in Jiangsu Province, China. However, the absence of detailed information on age, breed, sex distribution, and mortality rates represents a limitation of this study.

Our findings demonstrated that isolates from diseased goats were predominantly toxinotype D, followed by types A, C, and F, whereas isolates recovered from fecal and rumen samples of healthy goats were largely type A. This distribution is consistent with the well-established role of *C. perfringens* type A as a common commensal inhabitant of the gastrointestinal tract in animals and humans. Importantly, the detection of toxinotype D in symptomatic or deceased goats alone does not confirm enterotoxemia, as multiple infectious or non-infectious factors may contribute to similar clinical manifestations. Accordingly, this study focused on molecular characterization and AMR profiling rather than establishing direct causality between toxinotype and disease.

### Emerging and rare toxinotypes in goat populations

Enterotoxemia caused by *C. perfringens*, particularly toxinotype D, remains a leading cause of sudden death in goats worldwide. Experimental evidence has also confirmed goats as natural hosts for toxinotype C strains [28]. Toxinotype F (formerly classified as type A with *cpe*) has been infrequently reported in goats, sheep, and dogs [6, 29]. Notably, this study is among the first to document the presence of toxinotype G in goats from Jiangsu Province and may represent one of the earliest reports from China. Although toxinotype G was not detected in clinical cases, this observation aligns with findings from a large-scale Italian study analyzing 632 isolates over a 15-year period [30]. These results suggest that unidentified virulence determinants may play a role in the pathogenic potential of toxinotype G and warrant further investigation in well-characterized clinical cases.

### Phenotypic AMR patterns

Phenotypic antimicrobial susceptibility testing revealed that *C. perfringens* isolates from both healthy and diseased goats exhibited high resistance to kanamycin, neomycin, trimethoprim–sulfamethoxazole, and gentamicin, while remaining largely susceptible to β-lactam antibiotics, including penicillin, ampicillin, piperacillin, cefazolin, cefoperazone, and vancomycin. These resistance patterns may reflect inappropriate or excessive antimicrobial use for therapeutic purposes, disease prevention, or growth promotion in goat production systems.

The observed resistance to aminoglycosides is not unexpected, as strict anaerobes such as *C. perfringens* exhibit intrinsic resistance to these agents [31]. In contrast, the sustained susceptibility to β-lactam antibiotics is consistent with previous reports highlighting their bactericidal efficacy against *C. perfringens* and their clinical utility in severe infections [32–34]. Collectively, these findings underscore the need for targeted antimicrobial stewardship, routine surveillance, and vaccination strategies to mitigate risks to animal health, farm productivity, and public health.

### Genotypic resistance determinants and their epidemiological significance

Although AMR in goats has been reported previously, genotypic data on resistance determinants in *C. perfringens* remain scarce. In this context, the detection of resistance genes such as *qnrS* and *bla*_TEM_ provides novel molecular evidence that complements the phenotypic resistance profiles observed in this study. These findings suggest potential pathways for resistance dissemination within goat populations.

Quinolones, classified by the World Health Organization as critically important antimicrobials for human medicine [35, 36], warrant particular attention. The *qnrS* gene was detected in 44.12% of isolates from diseased goats but was absent in isolates from healthy animals. Similar resistance trends have been documented in gram-negative bacteria from goats with respiratory disease [37]. While quinolone resistance genes are typically plasmid-mediated in gram-negative bacteria [38, 39], they are often chromosomally encoded in gram-positive organisms [40]. These observations highlight a potential association between disease status and quinolone resistance and raise concerns regarding zoonotic and foodborne transmission within a One Health framework.

### Tetracycline resistance complexity in *C. perfringens*

Tetracyclines have been extensively used since the 1950s due to their broad-spectrum activity and favorable safety profile [41]. Resistance to tetracyclines remains the most prevalent resistance phenotype in *C. perfringens* [42], commonly mediated by *tetA(P)*, *tetB(P)*, and *tet(M)*, with *tetA(P)* frequently predominating [43–45]. In the present study, *tetA(P)* was the most prevalent tetracycline resistance gene, followed by *tetB(P)* and *tet(M)* [46]. However, phenotypic resistance levels were only moderate despite the high carriage of these genes.

This discrepancy may be explained by regulatory mechanisms such as reduced gene expression, gene silencing, mutational inactivation, or alternative resistance pathways, including efflux modulation and membrane permeability changes. These findings highlight the complex relationship between genotype and phenotype in tetracycline resistance and emphasize the importance of integrated molecular and phenotypic surveillance.

### Association between toxinotypes and AMR

Hierarchical clustering and heatmap analyses demonstrated significantly higher resistance to amino-glycosides, quinolones, and lincomycin among isolates from diseased goats compared to those from healthy animals. Similar trends have been reported in poultry, where isolates from necrotic enteritis cases showed elevated resistance to gentamicin, clindamycin, and virginiamycin [47]. While dominant toxinotypes and resistance patterns have been described in sheep and poultry [48–50], our findings indicate that goats harbor distinct toxinotype–resistance combinations.

Specifically, toxinotypes C, D, and F carried a broader spectrum of resistance genes, likely reflecting increased antimicrobial exposure in diseased animals [51]. Although previous studies have reported positive associations between virulence and AMR [52], our data showed that toxinotype D isolates harbored the highest prevalence of *qnrS*, *tetA(P)*, and *tetB(P)*, followed by toxinotypes F and C. Additionally, prior associations between the *netB* gene and increased resistance to virginiamycin further support the complex interplay between virulence and resistance [38, 53]. Nonetheless, antagonistic interactions between resistance and virulence plasmids have also been reported [43, 54], underscoring the multifaceted nature of these relationships.

### Implications and Future Perspectives

Overall, this study demonstrates a complex association between AMR and virulence in goat-associated *C. perfringens*. The inclusion of both healthy and diseased goats allowed for a more comprehensive understanding of commensal reservoirs and clinical strains, addressing a critical knowledge gap [52]. Continued large-scale epidemiological investigations integrating genomic, phenotypic, and ecological data are essential to elucidate the mechanisms driving resistance–virulence interactions and to support evidence-based strategies for disease control and antimicrobial stewardship in livestock systems.

## CONCLUSION

This study provides a comprehensive molecular and phenotypic characterization of *C. perfringens* circulating in goat populations in Jiangsu Province, China. From 404 samples, 94 *C. perfringens* isolates were identified, revealing substantial toxinotype diversity and a high burden of AMR. Toxinotype A predominated overall and among healthy goats, whereas toxinotype D was strongly associated with diseased or deceased animals, underscoring its clinical relevance in caprine enteric disease. Importantly, toxinotypes C, F, and G were also detected, with type G reported in goats in this region for the first time. Antimicrobial susceptibility testing demonstrated widespread resistance to aminoglycosides and trimethoprim–sulfamethoxazole, while most isolates remained susceptible to β-lactam antibiotics and vancomycin. Genotypic analysis confirmed the frequent presence of resistance determinants, particularly *tetA(P)*, *tetB(P)*, *lnu(B)*, *aph(3′)-III-F*, *qnrS*, and *bla*_TEM_, with significantly higher carriage of several genes in clinical isolates.

From a practical perspective, these findings have direct implications for goat health management and antimicrobial stewardship. The predominance of toxinotype D in clinical cases highlights the continued importance of targeted vaccination and early diagnostic surveillance for enterotoxemia. The high prevalence of resistance genes, including those associated with critically important antimicrobials such as quinolones, emphasizes the need to restrict indiscriminate antimicrobial use in goat farming. Moreover, the detection of resistance genes in isolates from healthy goats indicates that apparently normal animals can serve as reservoirs for AMR, facilitating dissemination within herds and posing potential zoonotic and food-chain risks under a One Health framework.

A major strength of this study lies in its large sample size, inclusion of both healthy and diseased animals, and the integrated analysis of toxinotypes, phenotypic resistance, and resistance genes. This combined approach provides a more complete understanding of the epidemiology of *C. perfringens* in goats than studies focused solely on clinical cases or toxin profiling alone. The use of hierarchical clustering further strengthened the interpretation of relationships between toxinotypes and AMR patterns.

Nevertheless, several limitations should be acknowledged. Detailed metadata on age, breed, sex, management practices, and prior antimicrobial exposure were unavailable, limiting risk-factor analysis. The use of surrogate disk diffusion breakpoints due to the lack of *C. perfringens*–specific CLSI criteria may also affect the precision of phenotypic resistance interpretation. Additionally, whole-genome sequencing was not performed, preventing deeper insights into plasmid structures, gene mobility, and virulence–resistance linkage.

Future research should focus on longitudinal surveillance across different production systems, integration of detailed farm-level antimicrobial usage data, and application of whole-genome sequencing to clarify the genetic context and transmission dynamics of resistance and toxin genes. Experimental studies exploring the functional relationship between toxin expression, antimicrobial exposure, and disease severity in goats would further enhance understanding and guide evidence-based control strategies.

In conclusion, this study demonstrates that goat-associated *C. perfringens* in Jiangsu Province exhibits marked toxin diversity and a considerable AMR burden. These findings reinforce the necessity for continuous surveillance, rational antimicrobial use, and integrated control measures to safeguard goat health, farm productivity, and public health.

## DATA AVAILABILITY

The supplementary data can be made available from the corresponding author upon request.

## AUTHORS’ CONTRIBUTIONS

ZH, WL, and JH: Conceived and designed the experiments. ZH, SS, and HW: Performed the experiments. ZH and WL: Drafted the manuscript. All authors have read and approved the final version of the manuscript.
